# Prevalence of cardiovascular risk factors in people with epilepsy

**DOI:** 10.1002/brb3.618

**Published:** 2016-12-20

**Authors:** Rosa Maria Vivanco‐Hidalgo, Alejandra Gomez, Antia Moreira, Laura Díez, Roberto Elosua, Jaume Roquer

**Affiliations:** ^1^ Neurovascular Research GroupFundació Institut Mar d'Investigacions Mèdiques (FIMIM)BarcelonaSpain; ^2^ Universitat Autònoma de Barcelona (UAB)BarcelonaSpain; ^3^ Neurology DepartmentParc de Salut MarBarcelonaSpain; ^4^ Cardiovascular Epidemiology and Genetics Research GroupFundació Institut Mar d'Investigacions Mèdiques (FIMIM)BarcelonaSpain

**Keywords:** antiepileptic drugs, cardiovascular risk factors, comorbidity, epilepsy

## Abstract

**Objectives:**

Epilepsy has been associated with cardiovascular comorbidity. This study aimed to assess the potential association between cardiovascular risk factors (CRFs), antiepileptic drugs (AEDs), and etiology.

**Material and Methods:**

A single‐center retrospective epilepsy cohort from the decade of 2004–2013 was assessed. Poisson regression models with robust variance were estimated to obtain CRF prevalence ratios (PR) according to AED prescription and etiology.

**Results:**

After excluding patients in the monotherapy group with vascular etiology or previous cardiovascular events, in the remaining 400 patients, enzyme‐inducer AEDs (EIAEDs), especially phenytoin (PHT), were associated with higher prevalence of dyslipidemia (PRa 1.77, *p* < .05), compared to valproic acid. No etiology was associated with higher prevalence of any CRF.

**Conclusions:**

Patients treated with EIAEDs, especially PHT, had higher prevalence of dyslipidemia.

## 1 Introduction

Epilepsy is one of the most prevalent chronic neurologic disorders (World Health Organization, 2015). Many people with epilepsy (PWE) receive lifelong prescription treatment.

Older antiepileptic drugs (AEDs), mainly enzyme inducers (EIAEDs), such as carbamazepine (CBZ), phenytoin (PHT), phenobarbital (PB), or primidone, may be associated with more adverse effects than newer AEDs. For example, they alter lipid profile, increasing serum cholesterol levels (Mintzer et al., 2009). However, there is a lack of information about the prevalence of dyslipidemia related to the use of AEDs, which would facilitate appropriate management to reduce the risk of vascular diseases. Another AED that has been related to vascular risk factors is valproic acid (VPA), which has been associated with metabolic syndrome (Kim & Lee, 2007). For these reasons, some authors have suggested starting new AEDs in newly diagnosed patients or changing EIAEDs if patients experience metabolism‐related effects (Brodie et al., 2013).

Higher prevalence of cardiovascular risk factors (CRFs) such as hypertension, diabetes, and high cholesterol has been reported in PWE, compared to general population (Elliott et al., 2009; Téllez‐Zenteno, Matijevic, & Wiebe, 2005). A recent review of comorbidity in epilepsy (Gaitatzis, Sisodiya, & Sander, 2012) suggested a causal association with hypertension and diabetes, both of which are risk factors for stroke. As cerebrovascular disease accounts for approximately 30% of newly diagnosed epilepsy in adults (Hauser, Annegers, & Kurland, 1993), that could reflect the causal association between these risk factors and epilepsy. Therefore, more information is needed to know if epilepsy itself—with an etiology other than vascular—is associated with CRFs.

Information about the prevalence of CRFs in PWE and their association with AED use or etiology is crucial to the management of vascular risk in these patients. The specific aim of the study was to assess whether there was an association between prescribed AEDs, epilepsy etiology, and classic CRFs.

## 2 Material and Methods

### 2.1 Study population

Patients included in the study were part of EPIVASMAR, a hospital‐based outpatient registry of Hospital del Mar. The outpatient clinic of this university medical center serves a population of 339,196 inhabitants in the city of Barcelona and is the referral center for PWE. EPIVASMAR is a retrospective register of PWE recruited from January 2004—when electronic medical records began to be available—to December 2013. The aims of the register were to describe CRF distribution and prevalence and cardiovascular events in PWE. A standardized data collection form was designed to collect demographic data, epilepsy characteristics (type, etiology, duration, AED treatment), date of inclusion in the register (regardless of the date of epilepsy diagnosis), CRFs at inclusion, and cardiovascular events. The register included only patients with a diagnosis of epilepsy according to the 2005 International League Against Epilepsy (ILAE) definition (Fisher et al., 2005). To identify cases for this study, electronic medical records of the patients attended in the outpatient clinic with a diagnosis of epilepsy were revised one‐by‐one by the principal investigator (RMVH). The international classification of diseases codification was not used because, at the time of data collection, it was not considered reliable. Etiology was assessed using the available test information (CT, MRI, EEG) and classified as genetic (epilepsy is the direct result of a known or presumed genetic defect(s) in which seizures are the core symptom of the disorder), structural/metabolic (there is a distinct other structural or metabolic condition or disease that has been demonstrated to be associated with an increased risk of developing epilepsy), and unknown cause (the nature of the underlying cause is as yet unknown; Berg et al., 2010). Treatment at inclusion was determined from prescriptions written by the general physician (GP) and neurologist. In this analysis, only participants older than 35 years were included. The register was approved by the local institutional ethics committee (Hospital del Mar‐IMIM 2014/5505/I) and followed national and international guidelines (Deontological Code, Declaration of Helsinki). Written informed consent was not required for this observational study. Patient information was anonymized and de‐identified prior to analysis.

### 2.2 Definition of cardiovascular risk factors

Diabetes was considered if a diagnosis had been recorded by a GP, the patient was under treatment, or fasting glucose was ≥126 mg/dl in two blood tests or ≥200 mg/dl or HbA1c >6.5% in any test performed close to the inclusion date. Hypertension was considered if patients were diagnosed by a GP, were under treatment, or had resting blood pressure measurements taken close to the study inclusion date of systolic blood pressure ≥140 mmHg or diastolic blood pressure ≥90 mmHg. Dyslipidemia was considered if patients were diagnosed by a GP, were under treatment, or fasting total cholesterol was ≥240 mg/dl or triglycerides ≥200 mg/dl (De Backer et al., 2003). Smoking was recorded as current, former, and never smokers, according to the electronic medical record.

### 2.3 Statistical analysis

A descriptive analysis of the variables of interest was performed calculating percentages in categorical and mean or median with corresponding standard deviations and interquartile range (depending on normal distribution) in quantitative variables. A bivariate analysis, using Chi‐squared for categorical variables, and *t* test or ANOVA for continuous variables, with their correspondent nonparametric test if needed, was performed to compare groups. Poisson regression models with robust variance were estimated to obtain a crude prevalence ratio (PRc) of CRF according to AEDs prescription and etiology. The EIAED group included CBZ, PHT, PB, and primidone. VPA was considered the reference drug as it has the lowest CRF PRc in the bivariate analysis. Adjusted PRs (PRa) for variables that were statistically significant in the bivariate analysis (age, sex, duration and etiology of epilepsy, and AED therapy) were then estimated in a multivariate model. Adding smoking did not change the magnitude of the PR. In order to get a parsimonious model, smoking was not included in the logistic regression due to the small number of smokers in the monotherapy cohort. To obtain stable PR estimates, only drugs prescribed in more than 50 patients and data from PWE receiving monotherapy were considered. First, CRF prevalence was compared between patients treated with EIAEDs and those taking non‐EIAEDs. The analysis was then repeated, defining the group of patients treated with VPA as the reference group and considering CRF prevalence according to each selected AED. A sensitivity‐adjusted analysis was also performed, excluding patients with vascular etiology, considered likely to be the main contributors to the CRF burden. Patients with cardiovascular events prior to receiving their epilepsy diagnosis were also excluded, for the same reason. A *p* value <.05 was set to indicate statistical significance. STATA v.12 statistical package (StataCorp. 2001. Statistical Software: Release 12.0; Stata Corporation, College Station, TX, USA) was used to perform the analysis.

## 3 Results

**Figure 1:**
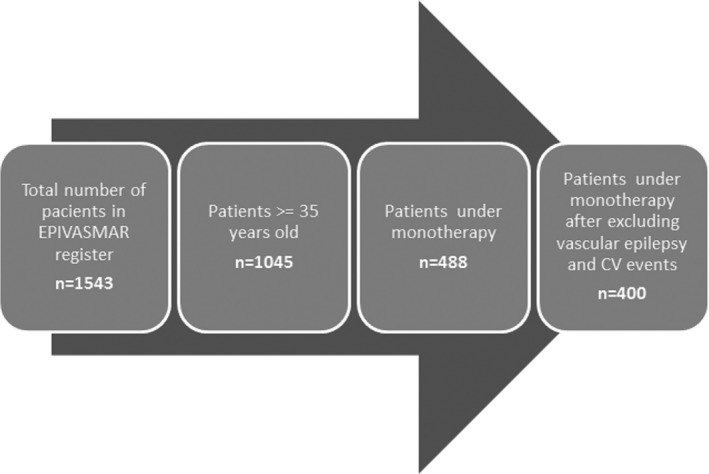
Flowchart showing patients included in the register and in the final analysis

Over a period of 10 years, 1,543 patients were included consecutively in the register, of which 1,045 were aged 35 years or older and included in the present analysis. The flowchart of patients included in the register and in the final analysis is shown in Figure 1.

Median age was 52.53 years (IQR: 42.60–66.11). The most prevalent etiology was structural/metabolic (519, 49.67%). Patients with genetic epilepsy were younger and had a lower prevalence of hypertension, dyslipidemia, and diabetes, compared to patients with structural and unknown etiologies (Table 1).

**Table 1:** Descriptive analysis of demographic and clinical data: total sample and by etiology

	Total (*N* = 1,045)	Genetic (*N* = 123, 11.77%)	Structural/metabolic (*N* = 519, 49.67%)	Unknown (*N* = 403, 38.56%)	*p* value
Median age in years at inclusion (IQR)	52.53 (42.60–66.11)	43.8 (39.8–54.04)	53.17 (43.66–67.37)	54.96 (42.94–69.24)	<.001
Women, *N* (%)	478 (45.74)	62 (50.41)	220 (42.39)	196 (48.64)	.091
Median epilepsy duration in years (IQR)	15.15 (1.975–33.85)	27.26 (15.23–37.22)	7.91 (0.86–28.84)	20.95 (4.92–37.11)	<.001
HTA, *N* (%)	342 (32.73)	15 (12.20)	193 (37.19)	134 (33.25)	<.001
DLP, *N* (%)	401 (38.37)	32 (26.02)	196 (37.76)	173 (42.93)	.003
DM, *N* (%)	137 (13.11)	7 (5.69)	83 (15.99)	47 (11.66)	.005
Smoking	228 (21.8)	28 (22.76)	121 (23.31)	79 (19.60)	.143

IQR, interquartile range; AED, antiepileptic drug; HTA, hypertension; DLP, dyslipidemia; DM, diabetes mellitus.

### 3.1 Cardiovascular risk factors and AEDs

Only patients under monotherapy (*n* = 488) and drugs prescribed in more than 50 patients were considered. Results from the bivariate analysis are summarized in Table 2. Multivariate analysis, adjusted by age, sex, etiology (genetic as reference), and epilepsy duration, showed that patients receiving EIAEDs had 36% higher prevalence of dyslipidemia, compared to those taking non‐EIAEDs. Only patients receiving PHT showed a significantly higher dyslipidemia prevalence, compared to patients under VPA (reference) therapy. After excluding vascular etiology and previous cardiovascular events, in the remaining 400 patients, EIAEDs (PRa 1.45, *p* < .05), and especially PHT (PRa 1.77, *p* < .05), were still associated with dyslipidemia (Table 3).

**Table 2:** Crude and adjusted prevalence ratio of cardiovascular risk factors according to the prescription of antiepileptic drugs, in patients under monotherapy

	HTA			DLP			DM		
	*N* (%)	PRc (95% CI)	PRa (95% CI)	*N* (%)	PRc (95% CI)	PRa (95% CI)	*N* (%)	PRc (95% CI)	PRa (95% CI)
Inducer vs noninducers AEDs									
Inducer AEDs (*N* = 185)	59 (31.89)	0.79 (0.62–1.02)	0.88 (0.69–1.11)	82 (44.32)	1.33 a (1.06–1.67)	1.36 a (1.07–1.72)	18 (9.73)	0.51 a (0.31–0.84)	0.73 (0.45–1.19)
Individual AEDs with VPA as reference group									
CBZ (*N* = 86)	21 (24.42)	0.76 (0.49–1.17)	0.71 (0.47–1.06)	31 (36.05)	1.41 (0.96–2.07)	1.28 (0.87–1.90)	6 (6.98)	0.44 (0.19–1.03)	0.65 (0.26–1.59)
PHT (*N* = 70)	31 (44.29)	1.37 (0.97–1.93)	1.04 (0.77–1.39)	37 (52.86)	2.06^a^ (1.47–2.91)	1.72^a^ (1.22–2.44)	8 (11.43)	0.72 (0.34–1.51)	0.65 (0.33–1.28)
LEV (*N* = 139)	69 (49.64)	1.54 a (1.16–2.03)	1.02 (0.81–1.30)	59 (42.45)	1.66^a^ (1.20–2.29)	1.33 (0.96–1.84)	32 (23.02)	1.45 (0.91–2.31)	0.96 (0.61–1.50)
VPA (*N* = 164)	53 (32.52)	1	1	42 (25.61)	1	1	26 (15.85)	1	1

Model adjusted by age, sex, etiology (genetic as a reference), and epilepsy duration. HTA, hypertension; DLP, dyslipidemia; DM, diabetes mellitus; PRc, prevalence ratio, crude; PRa, prevalence ratio, adjusted; CBZ, carbamazepine; PHT, phenytoin; LEV, levetiracetam; VPA, valproic acid.

a Statistically significant *p* < .05.

**Table 3:** Adjusted prevalence ratio of cardiovascular risk factors according to the prescription of antiepileptic drugs, in patients under monotherapy and without vascular epilepsy or cardiovascular events previous to diagnosis

	HTA		DLP		DM	
	*N* (%)	PRa	*N* (%)	PRa	*N* (%)	PRa
Inducer vs noninducers AEDs						
Inducer AEDs (155)	35 (22.58)	0.81 (0.58–1.13)	58 (37.42)	1.45 a (1.09–1.92)	11 (7.10)	0.77 (0.40–1.48)
Individual AEDs with VPA as reference group						
CBZ (77)	14 (18.18)	0.69 (0.40–1.18)	24 (31.17)	1.25 (0.78–2)	5 (6.49)	1.00 (0.33–3.07)
PHT (54)	18 (33.33)	1.07 (0.70–1.64)	25 (46.30)	1.77^a^ (1.15–2.73)	3 (5.56)	0.61 (0.19–1.97)
LEV (102)	41 (40.20)	1.12 (0.79–1.58)	37 (36.27)	1.36 (0.91–2.04)	17 (16.67)	1.32 (0.65–2.67)
VPA (143)	34 (23.78)	1	32 (22.38)	1	13 (9.09)	1

Model adjusted by age, sex, and epilepsy duration. In the case of hypertension, adjusted also by etiology (genetic as a reference). HTA, hypertension; DLP, dyslipidemia; DM, diabetes mellitus; PRa, prevalence ratio adjusted; CBZ, carbamazepine; PHT, phenytoin; LEV, levetiracetam; VPA, valproic acid.

a Statistically significant *p* < .05.

### 3.2 Cardiovascular risk factors and epilepsy etiology

**Figure 2:**
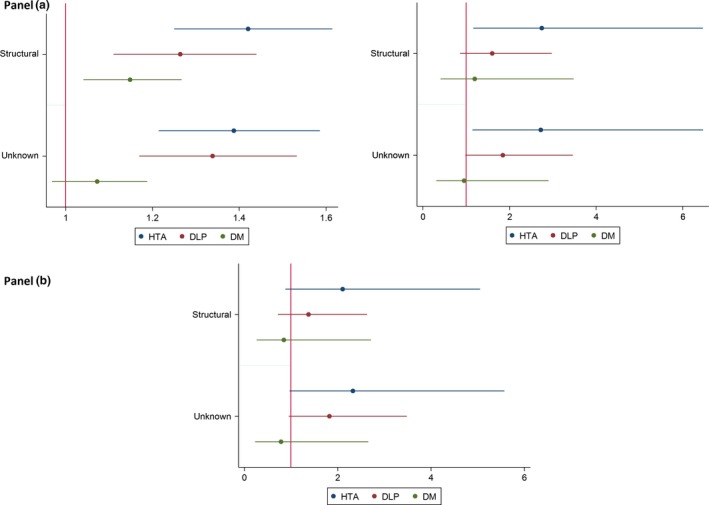
Results from the bivariate analysis. Panel (a): Crude and adjusted prevalence ratio of cardiovascular risk factors according to etiology and 95% confidence interval, in patients under monotherapy. Model adjusted by age, sex, epilepsy duration, antiepileptic drugs (AEDs). HTA, hypertension; DLP, dyslipidemia; DM diabetes mellitus. Panel (b): Adjusted prevalence ratio of cardiovascular risk factors according to etiology and 95% confidence interval, in patients under monotherapy and without vascular epilepsy or cardiovascular events previous to diagnosis. Model adjusted by age, sex, epilepsy duration, AEDs

Results from the bivariate analysis are shown in Figure 2, Panel a (left). Multivariate analysis adjusted by age, sex, AED monotherapy, and epilepsy duration showed a significant association between etiology and hypertension (Figure 2, Panel a, right). After excluding vascular etiology or previous cardiovascular events, the relation between hypertension and etiology lost its statistical significance, although a positive trend (PRa 2.11, 95% CI 0.88–5.04 for structural etiology and PRa 2.33, 95% CI 0.97–5.57 for unknown etiology) was observed (Figure 2, Panel b).

## 4 Discussion

Our study showed that dyslipidemia was the most frequent CRF and its presence was associated with EIAED therapy. A recent systematic review (Vyas et al., 2015) found that the three drugs mostly related to changes in lipid profile are PHT, CBZ, and VPA. In our case, EIAEDs—as a group—were associated with dyslipidemia diagnosis. However, only patients under PHT therapy had a higher prevalence of this CRF compared to those treated with VPA. No differences were found between patients treated with CBZ and those receiving VPA. Although we cannot discard the possibility that CBZ might have caused changes in cholesterol levels, the clinical significance is questionable, as any changes that occurred did not exceed the dyslipidemia definition threshold (De Backer et al., 2003). We consider this an important finding, as a systematic review (Vyas et al., 2015) reported changes in lipid profile (i.e., an increase in total cholesterol from 180 to 190 mg/dl) that did not necessarily result in a dyslipidemia diagnosis, making it difficult to assess prevalence of this CRF, and there was no consistency in association between VPA and total cholesterol levels. In our study, patients under VPA therapy were less affected by dyslipidemia than the rest of the population. We would speculate that the variety of lipid profile findings analyzed in the systematic review might correspond to differences not only in the study design (Grau et al., 2007) but also in the geographical region or dietary habits. Concerning new AEDs, we observed that the levetiracetam group had more CRFs, compared to patient groups taking other drugs (in monotherapy). However, after adjusting for several factors, the significance of the association disappeared. As this was a cross‐sectional study, that result might be explained by selection bias if patients had these CRFs before beginning levetiracetam therapy.

Another interesting result was the low prevalence of diabetes in the EIAEDs group. Inconsistent results in the literature make us cautious about the interpretation of this finding. In the past (Vaisrub, 1973), PHT was reported to be related to hyperglycemia and reduced insulin response in patients with glucose intolerance. However, we found no studies analyzing diabetes prevalence or showing that PHT, CBZ, or other EIAEDs cause diabetes mellitus. More studies are needed to compare diabetes prevalence (the least prevalent CRF) in this population to a general population without EIAEDs therapy.

Etiology has been reported to play a role in the relationship between epilepsy and CRF prevalence. In our analysis, the lowest prevalence of CRF was associated with genetic etiology. A recent review (Gaitatzis et al., 2012) reported high prevalence of CRFs in PWE and suggested that this is more likely a cause than a result of epilepsy, as they increase the risk of stroke, which is a cause of epilepsy. In our initial analysis, structural and unknown etiology were associated with higher prevalence of all three risk factors, compared to genetic etiology. In the multivariate analysis, however, this association remained only for hypertension. After excluding vascular epilepsy and previous cardiovascular events, this association disappeared. Thus, we concluded that our findings were related to vascular events previous to the epilepsy diagnosis and not to epilepsy itself. Despite this lack of statistical significance, the trend showing more hypertension in patients with structural and unknown etiology leads us to conclude that more studies are needed to clarify a finding that could be clinically relevant.

### 4.1 Limitations

As this was a cross‐sectional study, we could not establish any causal relationship between AEDs and CRF. We assumed a selection bias in treatment indication. For instance, levetiracetam is not related to drug interactions and therefore might have been prescribed in patients with more previous comorbidities. Another limitation was the sample size for the PR assessment. To give strength to the observed association and avoid mixing the effects of polytherapy, we selected only patients receiving monotherapy. The choice of VPA as a reference AED is another potential limitation, as VPA has been associated with metabolic syndrome (Kim & Lee, 2007). More recent studies have called the association into question, as this trend was not replicable in a recent population study (Rakitin et al., 2014) and did not differ from other AEDs such as CBZ in another report (Rakitin, Kõks, & Haldre, 2016). In our study, VPA had the lowest CRF PRc, and taking into account these recently published results, we considered that using it as the reference AED would not cause major confusion. Our analysis was not adjusted by body mass index or socioeconomic status, as we did not have access to this information. We do not consider this a major limitation, given the established relationship between these two indicators, because our reference population (Ciutat Vella and Sant Marti district) is very homogeneous in its socioeconomic profile (Barcelona Department of Statistics, 2016). In summary, treatment with EIAEDs, especially PHT, in PWE is associated with higher prevalence of dyslipidemia, but not with other CRFs. After excluding vascular etiology, there were no differences in CRF prevalence between etiologies, although a trend toward higher prevalence of hypertension was observed in structural and unknown etiologies, compared to genetic epilepsy. Further studies are needed to elucidate whether these results translate into a higher cardiovascular risk profile and therefore higher cardiovascular disease incidence in our study population.

## Conflict of Interest

Rosa Maria Vivanco‐Hidalgo had full access to all the data in the study and takes responsibility for the integrity of the data and the accuracy of the data analysis. The authors declare that there is no conflict of interest that could be perceived as prejudicing the impartiality of the research reported. We confirm that we have read the Journal's position on issues involved in ethical publication and affirm that this report is consistent with those guidelines.
